# Changes in the prevalence of dementia in Australia and its association with geographic remoteness

**DOI:** 10.1371/journal.pone.0289505

**Published:** 2023-08-02

**Authors:** Rezwanul Haque, Khorshed Alam, Jeff Gow, Christine Neville

**Affiliations:** 1 Department of Economics, American International University-Bangladesh (AIUB), Dhaka, Bangladesh; 2 School of Business, University of Southern Queensland, Toowoomba, Queensland, Australia; 3 Centre for Health Research, University of Southern Queensland, Toowoomba, Queensland, Australia; 4 School of Accounting, Economics and Finance, University of KwaZulu-Natal, Durban, South Africa; 5 School of Nursing and Midwifery, University of Southern Queensland, Toowoomba, Queensland, Australia; University of Oxford, UNITED KINGDOM

## Abstract

**Background:**

The exact prevalence of dementia in Australia is ambiguous. Australia is a vast continent with a small population, and 80% of the population live in five cities. This study explores recent changes in the prevalence of dementia. It also investigates geographic remoteness as a potential risk factor for developing dementia.

**Methods:**

Survey of Disability, Ageing and Carers (SDAC), a nationally representative database, was used to conduct this study. A total of 74,862 and 65,487 individuals from 2015 and 2018, respectively, were considered for this study. A multivariable logistic regression model was used to evaluate the association between dementia and geographic remoteness for older adults aged 65 years and over.

**Results:**

The results reveal that from 2015 to 2018, the prevalence of dementia among adults aged 65 years and older was higher in 2018 (5,229 per 100,000) than in 2015 (5,099 per 100,000). Significant geographical differences in the prevalence of dementia are observed among Australian adults, and this trend appears to be increasing. Furthermore, the unadjusted model revealed that, in 2015, older adults living in major cities had 1.29 (AOR: 1.29, 95% CI: 1.17–1.41) times higher odds of having dementia compared with their counterparts from outer regional and remote areas. In 2018, the adjusted model found that older adults living in major cities had 1.12 (AOR: 1.12, 95% CI: 1.01–1.25) times elevated odds of having dementia than their peers living in outer regional and remote areas.

**Conclusion:**

There is a rising prevalence of dementia in Australia. Further investigation is required to identify the causes of this increase. Increased public health initiatives should concentrate on behavioural characteristics and contextual environmental factors to ameliorate this trend.

## Introduction

Despite the projected increase of people living with dementia worldwide from approximately 55 million in 2020 to 139 million in 2050 [1], it is unknown how the prevalence of dementia will be in the future. According to the literature, there is no indication that the age-specific prevalence of dementia will change globally [2]. Nevertheless, the World Health Organization warned that estimates might not be reliable, particularly for older age groups [3]. For example, most research has found that estimates of the prevalence and incidence of dementia have likely decreased or remained stable over time in countries such as Nigeria, the Netherlands, the United Kingdom, Spain, Sweden, and the United States [4]. Japan, on the other hand, showed an increased prevalence of dementia [5, 6]. An earlier systematic review also concluded that the evidence on secular changes in dementia prevalence and incidence is equivocal, with contradictory findings in specific countries using various (and in some cases the same) datasets (e.g., the USA, the UK, and Sweden) [7]. Increasing education levels and implementing public health initiatives targeted at improving cardiovascular health are some changes to the primary causes of dementia risks that may help to lower prevalence rates. Conversely, a Western diet and an increase in sedentary behaviour may be responsible for a rise in dementia prevalence in several countries, including Japan [8].

Only a few previous studies have tried to determine trends in dementia prevalence in Australia. In 2010, Anstey and colleagues used information from two National Surveys of Mental Health and Wellbeing (NSMHW) conducted by the Australian Bureau of Statistics (ABS) and Dynamic Analyses to Optimising Ageing (DYNOPTA), a longitudinal study, to estimate the expected prevalence of dementia among individuals aged 65 years and older [9]. Later, these results were compared with estimates of the prevalence of dementia derived from meta-analyses of European studies. The Australian estimates found that the prevalence rates of probable dementia for those aged 65 to 69 years were, respectively, 3.78%, 6.22%, and 4% in three distinct surveys: DYNOPTA, NSMHW 1997, and NSMHW 2007. In these surveys, the prevalence of probable dementia among those aged 70 to 74 years was 5.16%, 9.09%, and 5.02%, respectively. DYNOPTA estimations were 10.63%, 16.32%, and 22.36% for the age groups of 75 to 79, 80 to 84, and 85 to 89, respectively. However, the NSMHW survey revealed less conformity with the meta-analyses, even though the DYNOPTA dataset was comparable to estimates obtained from meta-analyses, indicating that these are untrustworthy sources of information for forecasts. Since the population is expected to continue to age and it is assumed that the age-specific prevalence of dementia will not change, projection estimates for the future scale of dementia are mostly based on these assumptions. Only one recent study has looked at changes in dementia prevalence over time in Australia. However, it was a retrospective analysis of older adults who exclusively used long-term care [8]. According to the authors, age- and sex-standardized prevalence (95% confidence interval) of dementia decreased for those utilising long-term care from 50.0% (49.6, 50.5) in 2008 to 46.6% (46.0, 47.2) in 2014 and for those utilising home care from 25.9% (25.0, 26.5) in 2005 to 20.9% (20.2, 21.7) in 2014.

Geographic disparities in the incidence of non-communicable diseases are critical for public health interventions because they reveal illnesses with higher-than-average prevalence or ’hot spots’ [10]. Most Western European nations have within-country disparities in Alzheimer’s disease, according to a previous study [11]. However, a systematic review found that only a few studies have investigated the environmental causes of Alzheimer’s disease [12]. According to a previous systematic review and meta-analysis, those who have lived or currently reside in rural settings are more likely to have dementia [13]. This research was conducted in the United States, Canada, the United Kingdom, China, India, Italy, Nigeria, Turkey, Peru, and Mexico.

Prior studies have identified several environmental contributors as risk factors for dementia. For example, the concentration of air pollution in metropolitan areas may degrade cognitive function and raise the risk of Alzheimer’s disease [14, 15]. According to recent studies, being around green space may improve cognitive performance [16, 17], which is more prevalent outside of cities. Furthermore, even after controlling for air pollution, those who live close to major roads have been found to have an increased risk of dementia [18]. This evidence implies that dementia is more prevalent in urban than rural areas, notwithstanding the findings of most preceding studies. The Sax Institute’s 45 and Up study data for residents of New South Wales was the only Australian study to examine differences in Alzheimer’s disease risk based on geography [19]. The study found that the risk of Alzheimer’s disease was lower in rural and outlying locations compared to metropolitan cities using multilevel longitudinal analysis.

The precise prevalence of dementia is currently unknown in Australia because of multiple methodologies and the absence of a single reliable data source [20]. The Australian Institute of Health and Welfare provides data on dementia prevalence at the national level. Still, little is known regarding geographic remoteness and within-country differences, such as remote area living versus cities. Earlier studies on dementia prevalence in Australia were conducted using routinely collected aged care institutional data. However, this is the first study to look at trends in dementia prevalence in Australia using a nationally representative dataset that includes household and institutional care accommodation components. Investigating the relationship between geographic remoteness and dementia in Australia might also be prudent because of the significant geographic distances encountered. Therefore, this study intends to examine trends in dementia prevalence in Australia from 2015 to 2018 and to establish a link between dementia risk and geographic remoteness.

The present study is novel since it includes the distribution and comparison of dementia prevalence across Australian cities and rural-urban areas and the association between geographic remoteness and dementia using a nationally representative dataset for the first time.

## Methods

### Data source and settings

The current study uses microdata from the Survey of Disability, Ageing, and Carers (SDAC), a nationally representative household survey conducted by the ABS. A stratified, multi-stage area sample created by the ABS was used to choose the households. Computer-assisted personal interviews were used to gather data by trained interviewers. Instrument development and data collection methods adopted by the ABS are specified elsewhere [21, 22]. The survey was conducted in all states and territories, in both urban and rural locations. It included people who resided in private residences/households and institutions, including nursing homes, hospitals, and retirement communities. The SDAC includes data to assess the prevalence of disability and the need to aid persons with disabilities. It also provides a socioeconomic and demographic profile of individuals with disabilities, older adults, and caregivers compared to the general population. In addition, the dataset contains information about individuals with disabilities, long-term health conditions, and older adults.

### Study participants

**Fig 1:**
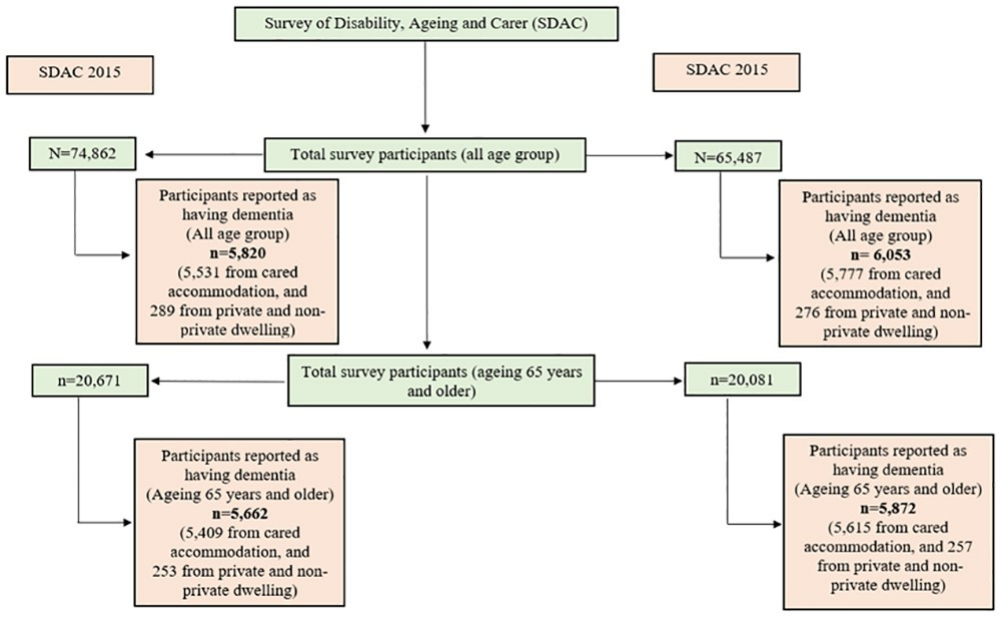
Distribution of study participants and year of survey.

Following comparable surveys conducted in 1981, 1988, 1993, 1998, 2003, 2009, 2012, and 2015, the 2018 SDAC is the seventh national survey. However, information concerning dementia, the primary variable of interest, is available only in 2015 and 2018. Therefore, this study considered data from these two rounds. The total sample comprised 74,862 and 65,487 individuals from private (e.g. houses and flats) and non-private dwellings (e.g. hotels and motels), and institutional cared accommodation establishments (e.g. hospitals and residential aged care) in 2015 and 2018, respectively. The study participants aged 65 years and older numbered 20,671 and 20,081 in 2015 and 2018, respectively. Distribution of the participants are displayed in Fig 1.

### Outcome variable

The primary focus of the current study is dementia, which was ascertained by self-reported and carer responses to the question "do you/persons have dementia?". The answer came from a binary choice of "yes" or "no". A follow-up question was to ask who else in the household had dementia. Dementia data was collected from both households and cared accommodation. The data obtained from the household component, which includes both private and non-private dwellings, is primarily derived from self-reported responses. Alternatively, a proxy, such as a carer, may provide the information in cases where the individual of interest is unable to respond on their own behalf. However, in the context of cared accommodation, the survey is not reliant on self-reporting, but rather is administered by carer who is obligated to document any chronic medical conditions. The scope of the gathered data was confined to the knowledge that can reasonably be anticipated from medical, nursing, and administrative records accessible to staff.

### Exposure variable

Geographic remoteness was the exposure of interest measured by the Accessibility Remoteness Index of Australia (ARIA). The ABS reclassified it into the following categories: i) "major city," (ii) "inner regional area," (iii) "outer regional," (iv) "remote", and (v) "extremely distant" [23]. Due to the small numbers in each group, individuals from "outer regional," "remote," and "very remote" areas in the SDAC dataset were combined into one category as "outer regional or remote area."

### Confounders

The SDAC’s cared-accommodation component collected limited data than the household component since some topics were either unsuitable for proxy data collection or irrelevant to people living in cared-accommodation [24]. Thus, this study could not sustain all potential confounders to conduct a complete case analysis because most data on people with dementia came from care accommodation. Covariates included in this study were age (65–69, 70–74, 75–79, 80–84, and 85 years or older), sex (male and female), and country of birth (Australia, English-speaking countries, and non-English-speaking countries).

### Estimation strategies

The current study uses Basic Confidentialised Unit Records Files from the 2015 and 2018 datasets for cross-sectional analysis. A weighted percentage was used to ensure that the individual estimate conforms to an independently determined distribution of the Australian population. The STATA command "svy set" was utilised in the analysis to handle the intricate survey design.

The characteristics of the study subjects have been compiled as frequency (n) and weighted percentage (%) with 95% confidence intervals (CIs). The chi-square test was used to examine the bivariate correlation between the primary variable of interest and covariates associated with the outcome variable. Only those predictors with a statistically significant level of 5% or less in the bivariate analysis were included in the adjusted model.

Multivariable logistic regression models examined the association between dementia and geographic remoteness. The logistic regression results were expressed as adjusted and unadjusted odds ratios (ORs) with 95% CIs, and a P-value at <0.05 level was found to be statistically significant. STATA 16 (Stata Corp LLC) was used to conduct the analysis, including cross-tabulation, regression, and summary statistics.

## Results

**Fig 2:**
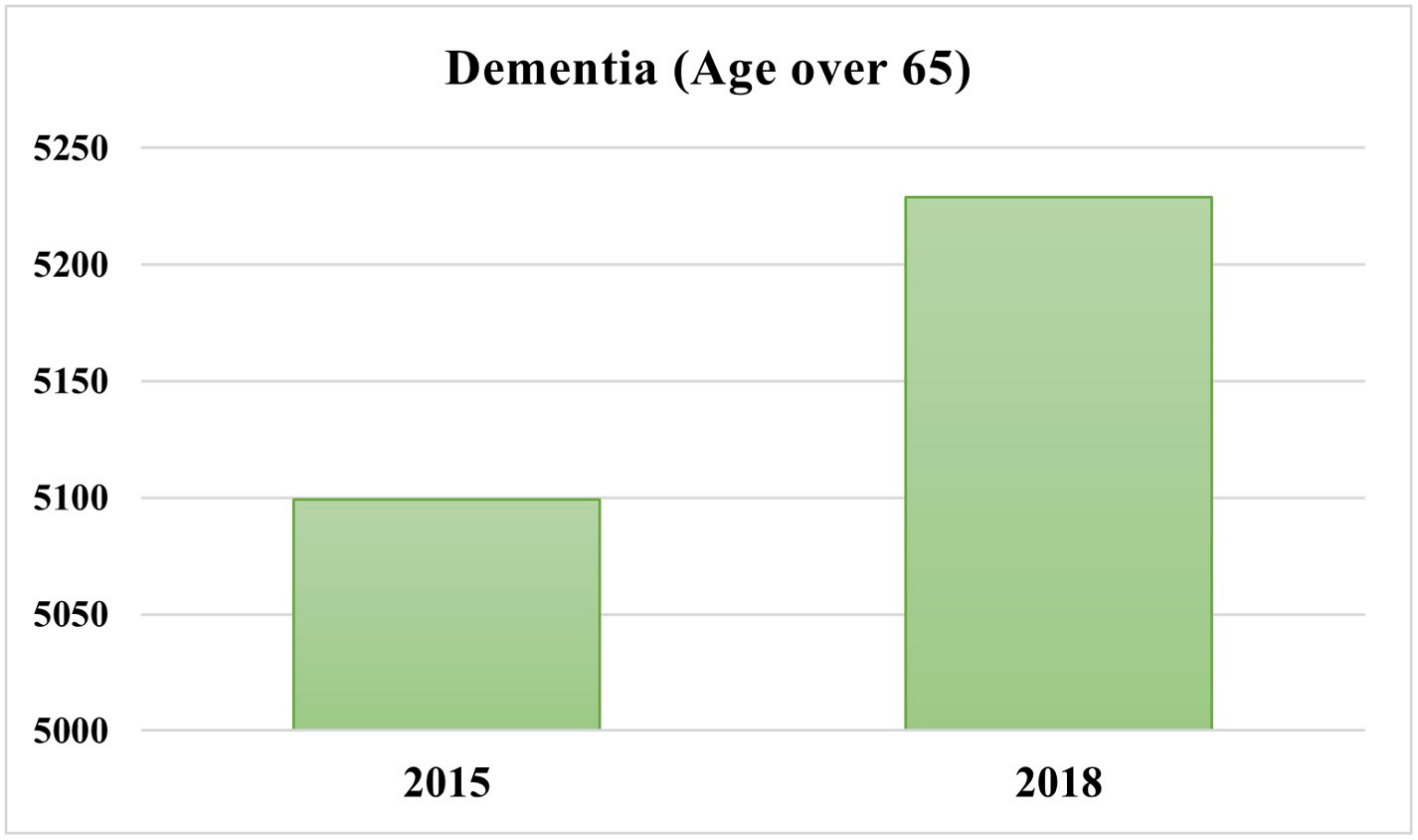
Changes in prevalence of dementia (per 100,000) in Australia, 2015–2018.

Fig 2 displays the changes in the prevalence of dementia for older Australians from 2015 to 2018. Fig 2 also shows that the prevalence rate of dementia among people aged 65 years and older increased from 5,099 per 100,000 in 2015 to 5,229 per 100,000 in 2018.

**Fig 3:**
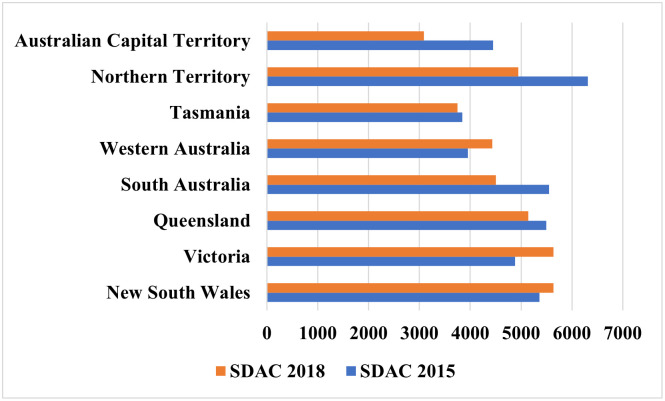
Changes in the prevalence of dementia (per 100,000) by state, 2015–2018.

Fig 3 illustrates the state-wise change in the prevalence (overall) of dementia in Australia. New South Wales, Victoria, and Western Australia observed an increased prevalence of dementia from 2015 to 2018. Victoria experienced a noticeable increase in the prevalence of dementia from 4,881 per 100,000 in 2015 to 5,637 per 100,000 in 2018. However, during the study period, South Australia, Tasmania, Northern Territory, Queensland and the Australian Capital Territory showed a decreasing trend in dementia prevalence.

**Fig 4:**
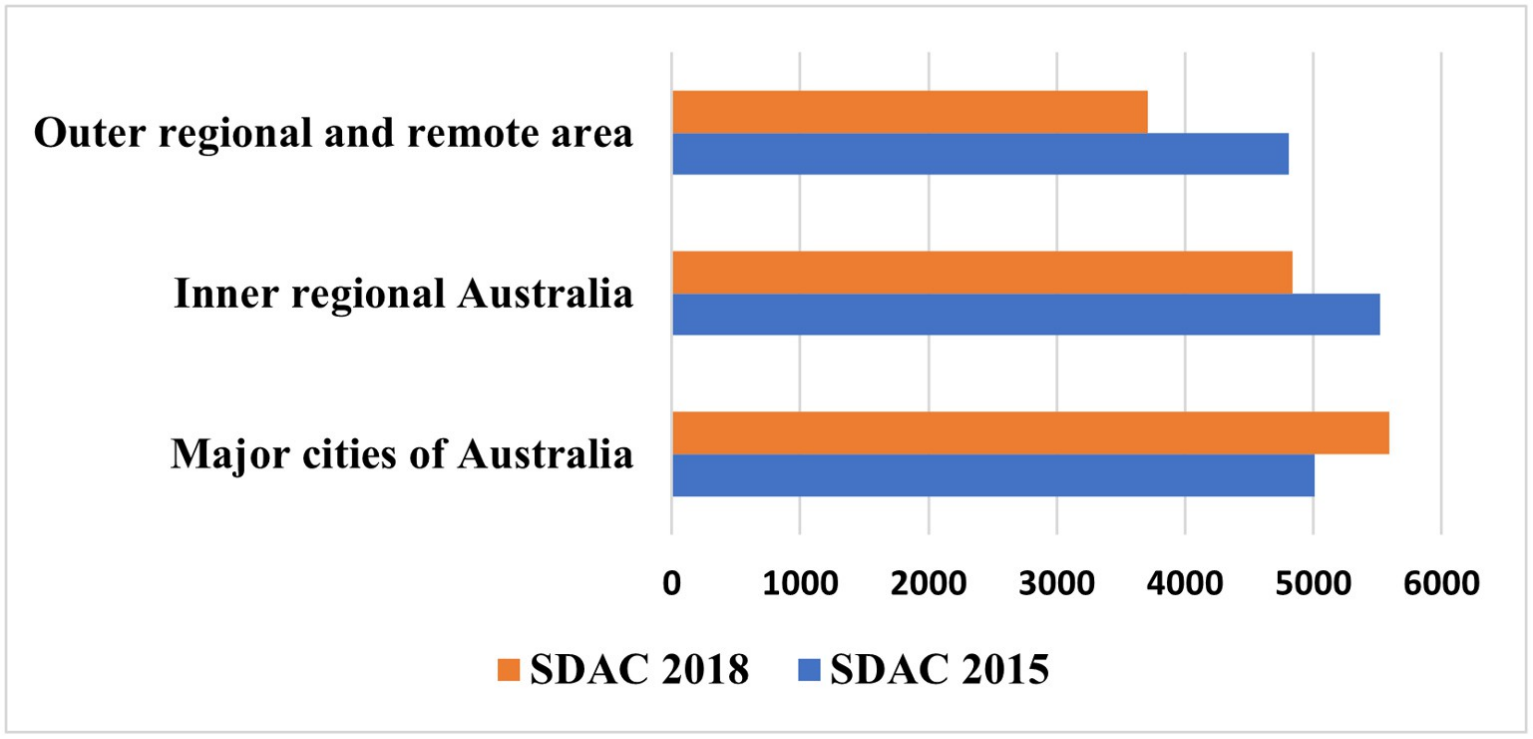
Changes in the prevalence of dementia (per 100,000) by geographic remoteness in Australia, 2015–2018.

Fig 4 demonstrates the changes in the prevalence (overall) of dementia from 2015 to 2018 by geographic remoteness. A substantial increase in the prevalence of dementia in major cities has been observed (from 5,010 in 2015 to 5,590 in 2018 per 100,000). Fig 4 also reveals that dementia among people living outer regional and remote areas dropped from 4,810 to 3,760 per 100,000 between 2015 and 2018.

Table 1 shows the changes in the prevalence of dementia in Australia by age and gender from 2015 to 2018. The overall prevalence of dementia rose from 0.84% to 0.89%. In addition, the prevalence of dementia among Australians aged 65 years and older increased from 5.10% to 5.23%. In both years, the largest prevalence was observed among individuals aged 85 years and older. Male prevalence of dementia increased while female prevalence decreased among older Australians.

**Table 1:** Weighted dementia prevalence by age and sex in Australia.

	SDAC 2015		SDAC 2018	
	n	% (95% CI)	n	% (95% CI)
Dementia prevalence (overall)	74,862	0.84 (0.78–0.89)	65,487	0.89 (0.82–0.96)
Dementia prevalence (age 65 years and older)	20,671	5.10 (4.76–5.46)	20,081	5.23 (4.85–5.64)
**Dementia prevalence by Age**				
*Below 65 years*	54,191	0.07 (0.05–0.09)	45,406	0.07 (0.04–0.10)
*65–69 years*	3,823	0.85 (0.60–1.22)	3,406	0.87 (0.59–1.28)
*70–74 years*	3,135	2.20 (1.67–2.89)	3,357	1.99 (1.49–2.66)
*75–79 years*	2,972	3.90 (3.21–4.72)	2,854	5.29 (4.36–6.39)
*80–84 years*	3,183	8.81 (7.58–10.21)	3,056	8.02 (6.81–9.42)
*85 years and older*	7,558	19.07 (17.52–20.73)	7,408	19.83 (17.95–21.86)
**Dementia prevalence by Sex (overall)**				
*Male*	34,987	0.65 (0.57–0.73)	30,302	0.77 (0.68–0.88)
*Female*	39,875	1.02 (0.94–1.11)	35,185	1.00 (0.91–1.10)
**Dementia prevalence by Sex (age 65 years and older)**				
*Male*	8,033	4.12 (3.65–4.65)	7,831	4.74 (4.17–5.38)
*Female*	12,638	5.96 (5.50–6.45)	12,250	5.66 (5.18–6.18)

Table 2 describes the socio-demographic characteristics of older adults with dementia that changed between 2015 and 2018. In both years, around 47–48% of older adults with dementia was found in the age group of 85 years and older. Among older adults with dementia, the proportion of women was found higher compared to men (62.33% vs 37.67% in 2015 and 57.54% vs 42.46% in 2018). The percentage of older adults with dementia climbed Australia’s major cities from 65.97% in 2015 to 71.78% in 2018, whereas it decreased in inner regional, outer regional, and remote areas during the study period. In comparison with 2015, persons with dementia who were born in Australia decreased in 2018; however, at the same time, the prevalence of dementia among those who were born in either English-speaking (except Australia) or other non-English speaking countries showed substantial increases.

**Table 2:** Weighted sample characteristics of participants reported as having dementia (aged 65 years and older).

	SADC 2015			SADC 2018		
	Dementia (n = 5,662)			Dementia (n = 5,872)		
	n	% (95% CI)	P Value	n	% (95% CI)	P Value
**Age**						
*65–69 years*	161	5.41 (3.82–7.61)	<0.001	186	5.11 (3.51–7.39)	<0.001
*70–74 years*	302	10.48 (8.09–13.46)		398	9.98 (7.56–13.07)	
*75–79 years*	603	13.59 (11.33–16.22)		644	18.25 (15.30–21.62)	
*80–84 years*	1,076	21.61 (18.85–24.65)		1,119	19.05 (16.37–22.05)	
*85 years and older*	3,520	48.92 (45.52–52.32)		3,525	47.60 (43.78–51.46)	
**Sex**						
*Male*	1,710	37.67 (34.30–41.17)	<0.001	1,875	42.46 (38.81–46.19)	<0.001
*Female*	3,952	62.33 (58.83–65.70)		3,997	57.54 (53.81–61.19)	
**Accessibility and remoteness index**						
*Major cities in Australia*	3,787	65.97 (62.62–69.17)	<0.001	4,111	71.78 (68.12–75.16)	<0.001
*Inner regional Australia*	1,265	23.21 (20.38–26.30)		1,182	20.92 (17.95–24.23)	
*Outer regional and remote area*	610	10.82 (8.89–13.10)		579	7.31 (5.41–9.79)	
**Country of birth**						
*Australia*	3,797	65.76 (62.48–68.90)	<0.002	3,728	58.65 (54.71–62.78)	<0.001
*Other English Speaking Countries*	718	10.94 (9.20–12.96)		733	13.29 (10.83–16.22)	
*Non-English-speaking countries*	1,147	23.30 (20.45–26.41)		1,411	28.06 (24.65–31.85)	

Abbreviations: CI: Confidence Interval

Table 3 displays the unadjusted and adjusted multivariate logistic regression analyses for the association between dementia and geographic remoteness in 2015 and 2018. In 2015, although the adjusted model showed no significant association between dementia and geographic remoteness, the unadjusted model showed a significant association that older adults living in major cities had 1.29 (AOR: 1.29, 95% CI: 1.17–1.41) times higher odds of having dementia compared with their counterparts from outer regional and remote areas. In 2018, both unadjusted and adjusted models showed a significant association between dementia and geographic remoteness. The adjusted model revealed that, in 2018, older adults living in major cities had 1.12 (AOR: 1.12, 95% CI: 1.01–1.25) times elevated odds of having dementia compared with their peers living in outer regional and remote areas.

**Table 3:** Multivariate analysis for the adjusted association between dementia and geographic remoteness.

	SDAC 2015		SDAC 2018	
	n = 20,671		n = 20,081	
	UOR (95%CI)	AOR (95%CI)	UOR (95%CI)	AOR (95%CI)
**Age**				
*65–69 years*	1.0 (Reference)	1.0 (Reference)	1.0 (Reference)	1.0 (Reference)
*70–74 years*	2.42*** (1.99–2.95)	2.42*** (1.99–2.95)	2.33*** (1.94–2.79)	2.33*** (1.94–2.79)
*75–79 years*	5.79*** (4.83–6.94)	5.71*** (4.76–6.85)	5.04*** (4.25–5.99)	5.01*** (4.22–5.96)
*80–84 years*	11.62*** (9.76–13.82)	11.37*** (9.55–13.53)	10.00*** (8.48–11.8)	9.81*** (8.32–11.57)
*85 years and older*	19.83*** (16.83–23.37)	18.99*** (16.11–22.4)	15.72*** (13.46–18.34)	15.19*** (13–17.73)
**Sex**				
*Male*	1.0 (Reference)	1.0 (Reference)	1.0 (Reference)	1.0 (Reference)
*Female*	1.68*** (1.58–1.8)	1.23*** (1.14–1.32)	1.54*** (1.44–1.64)	1.18*** (1.1–1.26)
**Accessibility and remoteness index**				
*Major cities in Australia*	1.29*** (1.17–1.42)	1.06 (0.96–1.19)	1.20***(1.08–1.33)	1.12*(1.01–1.25)
*Inner regional Australia*	1.28*** (1.15–1.43)	1.11 (0.98–1.25)	0.99 (0.88–1.11)	0.99 (0.87–1.12)
*Outer regional and remote area*	1.0 (Reference)	1.0 (Reference)	1.0 (Reference)	1.0 (Reference)
**Country of birth**				
*Australia*	1.0 (Reference)	1.0 (Reference)	1.0 (Reference)	1.0 (Reference)
*Other English Speaking Countries*	0.89* (0.81–0.98)	0.97 (0.87–1.07)	0.95 (0.86–1.04)	0.97 (0.88–1.07)
*Non-English-speaking countries*	1.09* (1.01–1.18)	1.16*** (1.06–1.27)	1.32***(1.22–1.42)	1.27***(1.17–1.37)

Abbreviation: UOR: Unadjusted Odds ratio; AOR: Adjusted Odds Ratio; CI: Confidence Interval

P values:

***P< 0.001,

**P<0.01;

*P<0.05

## Discussion

Over the three-year study period, the results demonstrate variations in the prevalence of dementia in Australia. They showed a significant regional disparity in frequency and an overall upward trend in dementia. Secondly, using cross-sectional nationally representative data, it was revealed that dementia was associated with geographic remoteness and that individuals residing in major cities had a higher risk of developing the disease than those in outer regional and rural locations.

The results demonstrate that the prevalence of dementia increased in Australia from 2015 to 2018. This result is consistent with eight extensive population studies conducted in Japan between 1985 and 2012, where all causes of dementia prevalence among those aged 65 years and over were increasing which ranged from 5.6% to 11.3% [6]. However, a study in the United States of America found that cumulative hazard rates of dementia were 3.6, 2.8, 2.2, and 2.0 per 100 persons during the first (the late 1970s to early 1980s), second (late 1980s to early 1990s), third (late 1990s to early 2000s) and fourth (late 2000s to early 2010s) epochs, respectively [25]. Other studies in the United Kingdom [26], Sweden [27], and Spain [28] for men alone, have also found a decrease in the prevalence or incidence of dementia. In addition, another study in the Netherlands reported a reduction in the prevalence of dementia, albeit without statistically significance [29].

In contrast to our findings, earlier Australian research, along with those of the majority of high-income nations, showed a downward trend in dementia prevalence. For example, in one retrospective study, dementia prevalence among older adults aged 65 years and older who used long-term aged care services between 2005 and 2014 was investigated [8]. According to that study, aged care service users in Australia experienced a decline in age- and sex-standardised dementia prevalence, which fell from 50% in 2008 to 46.6% in 2014. However, earlier in 2010, Anstey and colleagues compared the prevalence rates of dementia based on meta-analyses from European research to the probable dementia prevalence rates from the two most important sources of population-based data in Australia [9]. The study concluded that the incidence of dementia doubles approximately every five years within the age range of 70 to 84 years. However, the rate of escalation decelerates beyond this age range.

The observed rising trends in dementia could be due to a variety of factors. Earlier studies considered only those individuals who accessed long-term care while this study employed a nationally representative SDAC dataset. The aged care assessments employed in earlier studies may have only captured around 80% of people with dementia, according to a prior study using Australian-linked health data [30]. Hence, it is possible that previous studies underreported dementia from an Australian perspective. The time frame of the analysis could be another factor. Earlier research used datasets from 2008 to 2016, but this analysis included data from 2015 to 2018.

Age is considered the most significant risk of dementia [31], and the percentage of people aged 65 years and older in Australia with dementia rose from 12.2% to 15.7% over the 20 years between 1998 and 2018 [32], this may be a contributing factor to the growing prevalence of dementia. Additionally, the prevalence of diabetes, high blood pressure, obesity, undernutrition, depression, and brain injuries have increased over time in Australia; this may be a factor in the rise in dementia rates [8]. Additionally, rising public knowledge of dementia may have made functional and cognitive deficits that could have previously been written off as ‘normal ageing’ now be included as dementia [6]. Recent research indicates that overall, 88% of Australians can recognise the symptoms of dementia from the written clinical vignettes [33], an increase from 82% in a study conducted a decade ago [34]. Dementia might become more prevalent if persons with a stroke or Transient Ischemic Attack (TIA) survive longer due to advancements in medical care [6].

This study results also revealed that older Australians living in major cities had a higher risk of dementia than those living in rural and outlying regions. In contrast to our findings, a prior study using cross-sectional data claimed that the rate and frequency of Alzheimer’s disease were greater in the countryside than in urban settings [13]. However, our results are consistent with a prior Australian study where the authors used multi-level longitudinal analysis and concluded that after adjusting for socio-demographic and geographic disadvantages as confounders, compared to rural and remote places, major cities had a higher risk of Alzheimer’s disease [35]. In addition, the findings of this study are consistent with research from Spain and the UK, which found that the prevalence of Alzheimer’s disease was lower in rural areas than in urban ones [36, 37]. In Australia, it was estimated that as of June 2020, two-thirds of older people (aged 65 years and older) lived in major cities (66%, 2.7 million) [38]; this is the most likely cause of the elevated incidence of dementia in metropolitan areas. Another potential explanation for the higher risk of having dementia in urban areas might be environmental factors. For example, earlier research identified chronic noise exposure, air pollution, and a paucity of green space as probable risk factors for cognition reduction, which are more prevalent in metropolitan areas [16–18, 39, 40].

It is possible that people residing in urban areas have a greater understanding of dementia due to their higher levels of education and income, which could explain the higher rates of people reported as having dementia in major cities compared to inner regional and outer regional areas. Prior research found that a higher level of education is a predictor of increased dementia knowledge [41–43]. In Australia, there are educational disparities, as 72% of students in metropolitan areas, 65% of students in regional areas, and 36% of students in remote areas complete secondary school [44]. Moreover, people residing in major cities were more likely to hold a bachelor’s degree or higher (36%) than those residing in inner regional areas (21%), outer regional areas (19%), and remote and very remote areas (18%) [45]. Furthermore, people with higher incomes have greater access to dementia-related information and, thus, increased dementia knowledge [41]. In 2017–18, the average weekly income and average household net worth of Australians living outside of capital cities were 19% and 30% lower, respectively, than those residing in capital cities [46].

The study findings have critical public health ramifications because they showed a statistically significant link between dementia and living in cities. Governments in Australia, particularly those at the federal, state, territorial, and local levels, can play an essential role in developing and delivering dementia-specific policies and services. Additionally, state and territory governments could provide additional funding for vital services such as memory clinics, geriatric assessments and home visits for older adults, services for older adults’ mental health, hospital-to-residential aged care transition services, and assistance for those who are exhibiting behavioural and psychological signs of dementia. These policies align with findings of the 2019 Aged Care Quality and Safety Royal Commission [47].

Prior research indicates that green spaces and increasing the number of urban trees could lower dementia risk [19, 48] by encouraging physical activity, social interaction, and network building while simultaneously reducing exposure to air pollution. Councils could develop standalone urban forest strategies or integrate the conservation of urban forests into municipal strategic planning statements to ensure that residents and communities have healthier environments. For Greater Sydney councils, the NSW Government has provided updated tree canopy data (2019) which can be treated as a foundation for developing urban forest initiatives [49].

The main strength of this study is the use of the SDAC dataset, a nationally representative sample of the population [50], in examining Australia’s dementia prevalence. Much of the prior information regarding dementia prevalence in Australia was derived from studies conducted using routinely collected aged care assessment data. However, individuals with dementia residing at-home were ignored in earlier research. This is the first study in Australia that used a nationally representative dataset that covers households and cared accommodation components to examine changes in the prevalence of dementia. Further, this study considers a new geographic characteristic, geographic remoteness, to check its association with dementia using a nationally representative dataset.

It is essential to consider the study’s limitations. First, the utilisation of self-report or proxy-reporting poses a significant challenge, especially in cases where an individual’s cognitive abilities are compromised, leading to a prolonged and uncertain diagnosis process. In addition, the presence of stigma may cause individuals to be hesitant to identify themselves. The SDAC may lead to underestimating mild and moderate dementia within the household population. Identifying individuals with dementia, especially at advanced ages, presents additional challenges due to co-occurring health conditions that obscure the symptoms of dementia. The aforementioned challenges are likely to have an impact on the information obtained through self-administered or proxy-based questionnaires. Although the cared accommodation component of the SDAC is considered as a strength of Australian data, it lacks comprehensive information regarding the socio-demographic characteristics of its residents. Furthermore, within the realm of cared accommodation, there may exist obstacles to the acquisition of a dementia diagnosis. However, the implementation of the Aged Care Funding Instrument (ACFI) within residential aged care has the potential to enhance identification practices in this sector, thereby leading to enhancements in the cared accommodation component of the SDAC [51]. Second, due to the cross-sectional research design, this study was unable to identify the causal pathways between dementia and geography. Third, the adjusted model of dementia and geographic remoteness had to be restricted to accessible confounders to provide a complete case analysis because most of the dementia data were collected from aged care accommodations and information on several socio-economic characteristics were not available. After considering these constraints, the results imply that future research should focus on prospective longitudinal studies to explore further the prevalence and the role of geography over time.

## Conclusion

Using a nationally representative data set, this study has revealed changes in the prevalence of dementia among Australian older adults by examining individual and geographical characteristics. This study has shown substantial differences in dementia prevalence among Australians during the study period. It was shown that there is a significant geographic disparity in the prevalence of dementia in Australia. Estimates from multivariable logit models support the finding that people who live in large cities have a greater risk of dementia than those living in outer regional and remote areas. Public health initiatives that are geographically focused and health education that encourages awareness and a healthy lifestyle could aid in halting Australia’s rising dementia rate. This study adds to the scant body of knowledge about regional variations in dementia prevalence in Australia.

## Abbreviations

ABS: Australian Bureau of Statistics
AOR: Adjusted Odd Ratio
CI: Confidence Interval
DYNOPTA: Dynamic Analyses to Optimise Ageing
NSMHW: National Surveys of Mental Health and Wellbeing
SDAC: Survey of Disability, Ageing, and Carers
WHO: World Health Organization
